# *Staphylococcus aureus* is able to generate resistance to novel lipoglycopeptide antibiotic gausemycin A

**DOI:** 10.3389/fmicb.2022.963979

**Published:** 2022-09-29

**Authors:** Darya V. Poshvina, Diana S. Dilbaryan, Sergey P. Kasyanov, Vera S. Sadykova, Olda A. Lapchinskaya, Eugene A. Rogozhin, Alexey S. Vasilchenko

**Affiliations:** ^1^Laboratory of Antimicrobial Resistance, Institute of Environmental and Agricultural Biology (X-BIO), Tyumen State University, Tyumen, Russia; ^2^A.V. Zhirmunsky National Scientific Center of Marine Biology, Vladivostok, Russia; ^3^Gause Institute of New Antibiotics, Moscow, Russia; ^4^Shemyakin and Ovchinnikov Institute of Bioorganic Chemistry Russian Academy of Sciences, Moscow, Russia

**Keywords:** gausemycins, lipoglycopeptides, *Streptomyces roseoflavus* INA-Ac-5812, antibiotic resistance, *Staphylococcus aureus*

## Abstract

Gausemycin A is the first member of the novel lipoglycopeptides family produced by *Streptomyces roseoflavus* INA-Ac-5812. Gausemycin A has a pronounced bactericidal activity against methicillin-resistant *Staphylococcus aureus*. However, the ability of *S. aureus* to be resistant to gausemycin A has not been investigated yet. Using serial passaging, we have obtained the resistant variant *S. aureus* 5812R, which is 80 times more resistant compared to the parent strain. Susceptibility testing of *S. aureus* 5812R revealed the acquisition of cross-resistance to daptomycin, cefazolin, tetracycline, and gentamicin, while the resistance to vancomycin, nisin, and ramoplanin was absent. Whole genome sequencing revealed single nucleotide polymorphism (SNP) and deletions in *S. aureus* 5812R, among which are genes encoding efflux pump (*sepA*), the two-component Kdp system (*kdpE*), and the component of isoprenoid biosynthesis pathway (*hepT)*. Phenotypically, *S. aureus* 5812R resembles a small-colony variant, as it is slow-growing, forms small colonies, and is deficient in pigments. Profiling of fatty acids (FA) composition constituting the cytoplasmic membrane of *S. aureus* 5812R revealed the prevalence of *anteiso*-branched FA, while straight FA was slightly less present. The evidence also showed that the gausemycin A-resistant strain has increased expression of the *cls2* gene of the cardiolipin synthase. The performed checkerboard assay pointed out that the combination of gausemycin A and ciprofloxacin showed a synergistic effect against *S. aureus* 5812R.

## Introduction

Antimicrobial resistance (AMR) poses a serious global threat to human, animal, and environmental health. The emergence and rapid spread of pathogenic microorganisms with multidrug resistance (MDR) to antibiotics, observed in recent decades, is especially dangerous (Bhullar et al., 2012; Perry et al., 2016; Aslam et al., 2018). The list of the main Gram-positive pathogens includes methicillin-resistant *Staphylococcus aureus* (MRSA) and vancomycin-resistant *S. aureus* (VRSA), since they play a leading role in the etiology of a wide range of community-acquired and hospital-acquired human infections (DeLeo and Chambers, 2009; Watkins et al., 2012; Rapacka-Zdonczyk et al., 2019). Most of the new antibiotics discovered over the past 30 years have shown activity mainly against Gram-positive bacteria (Lewis, 2020), in this regard, antibiotic resistance among Gram-positive bacteria is an alarming phenomenon.

Antimicrobial peptides (AMPs) of various origins are considered a powerful weapon against a resistant form of pathogens (Sharma et al., 2014; Singh et al., 2014; Lum et al., 2015; Raman et al., 2015; Lyu et al., 2016; Gwynne and Gallagher, 2018). Non-ribosomal peptide antibiotics (NRPs) attract considerable interest from the standpoint of their therapeutic potential (Li, 2018). Glycopeptide vancomycin is a conventional antibiotic with a long clinical history originating from the 1950s, and the spread of vancomycin-resistant bacterial strains occurred in 1980s (Wilhelm and Estes, 1999). Lipopeptide daptomycin was discovered in 1985 and approved by the US Food and Drug Administration (FDA) in 2003 (Mishra et al., 2012). Although daptomycin has become available in medical practice relatively recently, daptomycin-resistant isolates have already widely appeared (Bayer et al., 2013). Ramoplanin is a glycolipodepsipeptide, which contains a carbohydrate molecule and a fatty acid, both attached to a peptide core. Ramoplanin was discovered in the 1980s (Cavalleri et al., 1984), and the last available clinical trial status is phase III for VRE treatment (de la Cruz et al., 2017). Ramoplanin-resistant *S. aureus* strain was obtained *in vitro* in 2010 (Schmidt et al., 2010).

Recently, new lipoglycopeptides have been revealed among metabolites of actinomycete *Streptomyces roseoflavus* INA-Ac-5812. These lipoglycopeptides were firstly described in 2016 (Lapchinskaya et al., 2016), and consequently named gausemycins A and B (Tyurin et al., 2021). Gausemycin A is a macrocyclic peptide containing 14 amino acids, where tyrosine is glycosylated with arabinose, which makes gausemycin A unique among natural peptide antibiotics (Tyurin et al., 2021). The closest structurally related compounds are depsipeptides (daptomycin, taromycins, and cadazides) and cyclopeptides (amphomycin, rumycins, and malacidins) (Tyurin et al., 2021). Gausemycin A demonstrates rapid membrane disturbing activity against planktonic *S. aureus*. Gausemycin A was able to inhibit biofilm-embedded staphylococcal cells, while daptomycin was inactive (Vasilchenko et al., 2020).

Thus, the above-mentioned properties of gausemycin A make it an alternative to existing antibiotics for the treatment of infectious diseases caused by *S. aureus*. However, before a new substance will move to preclinical investigations, it is necessary to evaluate its ability to generate antibiotic-resistant bacterial variants (Parachin and Franco, 2014).

In this regard, this work aims to evaluate the ability of model strain *S. aureus* FDA209P to develop resistance to new lipoglycopeptide gausemycin A, and recognize the genomic and phenotypic changes occurring in the *S. aureus* population under the treatment.

## Materials and methods

### Bacterial culture and chemicals

*Staphylococcus aureus* FDA209P (gausemycin A-susceptible) was used in this study. Daptomycin, vancomycin, ramoplanin, nisin, cefazolin, tetracycline, gentamicin, ampicillin, and ciprofloxacin were purchased from Sigma-Aldrich (US).

Gausemycin A was obtained as described (Vasilchenko et al., 2020). Briefly, the culture medium after *S. roseoflavus* INA-Ac-5812 fermentation was applied to Amberlite XAD2 (Serva, UK) low-pressure column, and an unbound liquid was collected and further extracted by *n*-butanol in a ratio of 1:1 (v/v). The obtained butanolic extract was fully evaporated and resolved in 70% water-ethanol. Gausemycin A isolation was made using semi-preparative high-performance liquid chromatography on XBridge BEH C_18_ column (10 x 250 mm) (Waters, Ireland). The target compound was detected at a wavelength of 364 nm, which is optimum for 4-l-chloro-kenyrenine amino acid residue. The purity of gausemycin A was confirmed by re-chromatography with an analytical reversed-phase HPLC column (XBridge BEH C18 column, 4.6 x 250 mm, Waters, Ireland) and absorbance was monitored at 214 nm.

### Determination of minimum inhibitory concentrations

Minimal inhibitory concentration (MIC) was determined as previously described in Fuchs et al. (2000), following the Clinical and Laboratory Standards Institute (CLSI) guidelines for broth microdilution MIC assay. The bacteria were incubated in a 96-well microtiter plate (Eppendorf, Germany) containing 90 μL of inoculum prepared in growth media at 10^6^ CFU/ml with 10 μL of twofold dilutions of the antibiotics. The results were evaluated after 24 h of cultivation at 37^0^C. The bacterial growth was assessed by scanning the absorbance data at 600 nm obtained by spectrophotometer (Multiscan GO, Thermo Fisher Scientific, US).

### Serial passage experiments

Gausemycin A-resistant variant was obtained by serial passaging as described previously (Pollard et al., 2012). Isolated colonies of *S. aureus* FDA209P were inoculated into Mueller-Hinton II broth (CaCl_2_ 50 mg/L) containing twofold dilutions of gausemycin A. The cultures were incubated at 37 °C with aeration for 24 h. After incubation, bacterial cells growing at the highest concentration of gausemycin A (below the MIC) were harvested and used for the next passage. The process was repeated for 20 passages. The dynamic of the developing resistance to gausemycin A was evaluated in comparison with daptomycin and ramoplanin. The experiments were performed in two independent series on separate days with three technical replicates each.

### Resistance stability testing

The overnight culture of *S. aureus* 5812R was sub-cultured in gausemycin A-free Muller-Hinton broth and incubated overnight at 37°C with shaking. The obtained culture was evaluated on susceptibility to gausemycin A in the MIC assay as described previously. Passaging in the drug-free medium was repeated 17 times. The experiments were performed in two independent series on separate days with three technical replicates each.

### Assessment of phenotypical changes in *S. aureus* 5812R-resistant strain

#### Quantification of staphylococcal pigment

*S. aureus* strains were grown for 24 h at 37°C on agarose Mueller Hinton broth with the addition of sheep's blood (Tubby et al., 2013). Cells were washed from plates and harvested by centrifugation (4,500 g, 15 min); then washed twice with phosphate-buffered saline (PBS) (Sigma-Aldrich, US).

Staphyloxanthin and carotenoids were extracted and quantified as previously described with slight modification (Zhang et al., 2018). Cultures of *S. aureus* were equilibrated for optical density (OD_600nm_ 0.1) and then subjected to 50% ethanol. Washed cells were re-suspended in 2 mL 50% ethanol, heated in ultrasonic bath at 55°C for at least 5 min while being stirred gently, until all visible pigment of bacteria was extracted and dissolved into ethanol. After that, the ethanol extract was cooled and centrifuged. Pigment content was then quantified by the absorbance profile of carotenoids as determined spectrophotometrically at an optical density of 450 nm (OD_450_). The experiments were performed in three independent series on separate days with three technical replicates each. The means plus standard deviations of the means of results from three independent series with three technical replicates are shown.

#### Atomic force microscopy

*S. aureus* strains were grown for 24 h at 37°C on agarose Mueller Hinton broth with the addition of sheep's blood (Tubby et al., 2013). Cells were washed from plates and harvested by centrifugation (4,500 g, 15 min); then washed twice with double distilled water.

Atomic force microscopy (AFM) was performed using Integra NT-MDT (NT-MDT, Russia) in the tapping mode. The microscopy investigation was carried out using the NSG01 cantilever (Tipsnano, Estonia) with a spring constant of ~5.1 N/m. Preliminarily, wide scanning of about 70 × 70 μm was conducted to produce a reference map of the sample surface in order to help localize cells. The scan size was then set with a sampling of 512 by 512 points and a scan rate of 0.5 Hz.

#### Growth rates and biochemical properties of studied strains

*S. aureus* 5812R and *S. aureus* FDA209P strains were incubated in a 96-well microtiter plate (Eppendorf, Germany) containing 10 μL of inoculum (10^7^ CFU/ml) and 90 μl of drug-free Muller-Hinton media. The dynamics of bacterial growth were assessed by scanning and plotting the absorbance data at 600 nm obtained by spectrophotometer (Multiscan GO, Thermo Scientific, US). The experiments were performed using two independent series with three technical replicates each.

The biochemical properties were assayed using the ID32 Staph strip (BioMerieux, France), which consists of a set of wells containing dried biochemical media for colorimetric tests.

#### Zeta potential measurement

The electrophoretic mobility (EPM) of bacterial cells was measured with a zeta potential analyzer (Litesizer 500, Anton Paar, Austria) before being converted to zeta potentials using the Helmholtz–Smoluchowski theory as described in Shireen et al. (2013). Prior to sample analysis, the electrodes were polarized using the test solution in the absence of cells under identical conditions to establish consistent sample conductance readings. Between each measurement, electrodes were rinsed with copious amounts of ethanol and double distilled water, followed by the test bacterial suspension. The assay was performed twice on separate days.

#### Permeabilization of the plasma membrane and microscopy

Membrane integrity was assessed using the fluorescent probes SYTO 9 and propidium iodide (PI) (LIVE/DEAD BacLight Bacterial Viability Kit, Molecular Probes, USA). The test strains were grown to the mid-log phase. The obtained cultures were centrifuged at 10,000 rpm for 10 min, the supernatant was discarded and the pellet was dispersed in 10 mM HEPES buffer supplemented with 1.25 mM Ca ^2+^ to an optical density of 0.2 (OD _600_) that corresponded to 10^8^ CFU/ml. Bacterial cells in 50 μl were transferred to a 96-well microtiter plate (Eppendorf, US). SYTO 9 fluorescence kinetics was measured using Fluoroscan Ascent FL plate reader (Thermo Scientific, US) at 485 nm excitation and 535 nm of emission wavelengths as described earlier (Vasilchenko et al., 2020). The assay was performed twice in three technical replicates.

Microscopy was performed using a Zeiss Axio Imager A2 fluorescent microscope (Carl Zeiss, Germany) equipped with filter sets useful for simultaneous viewing of SYTO 9 and PI stains.

#### Membrane lipid extraction, fractioning, and methylation

Fatty acid (FA) composition was determined using biomass of *S. aureus* FDA209P and *S. aureus* 5812R. Bacterial cultures were grown to the stationary phase in Muller-Hinton medium with CaCl_2_ at 37°C with shaking. Bacterial cells were harvested by centrifugation, washed twice with PBS, and lyophilized.

Lipid extraction was performed according to the Folch method (Folch and Lees, 1957). Chloroform: methanol (2:1, v/v) was added to each sample according to the original Folch procedure allowing for a CHCl_3_:MeOH:H_2_O ratio of 8:4:3 (v/v/v). Briefly, ice-cold methanol and chloroform were added to the sample. The suspension was vortexed occasionally to bring about physical mixing and the sample was incubated on ice for 30 min. After the addition of water, which was used to separate the aqueous and organic layers, the suspension was incubated on ice for additional 10 min. Samples were centrifuged at 2,000 rpm for 5 min at 4°C. The lower phase (organic) layer was transferred to a new tube. The aqueous layer was re-extracted with 1 mL of 2:1 (v/v) chloroform/methanol. The chloroform layers were combined for analysis. The aqueous layer was centrifuged at 2,000 rpm for 5 min at 4 °C and collected for metabolite analysis. Extracts were then dried under nitrogen.

Fatty acid methyl esters (FAME) were obtained by the treatment of the total lipids with 2% H_2_SO_4_ in MeOH in a screw-capped vial (2 h, 80°C) under Argon and purified by thin-layer chromatography development in benzene (Carreau and Dubacq, 1978). The gas chromatography (GC) analysis of FAME was carried out on Shimadzu GC-2010 chromatograph (Kyoto, Japan) with a flame ionization detector on an Equity-5 (Supelco, Bellefonte, PA, US) capillary column (30 m/0.25 mm ID), at 160 °C with a 2 °C/min ramp to 240°C that was held for 20 min. Injector and detector temperatures were 250 °C. FAME were identified by gas chromatography-mass spectrometry (GC–MS) using Shimadzu GCMS-QP5050A instrument (Kyoto, Japan) (electron impact at 70 eV) with an SPB-5 (Supelco, Bellefonte, PA, US) capillary column (30 m /0.25 mm ID). The GC–MS analysis of FAME was performed at 160°C with a 2°C/min ramp to 240°C that was held for 20 min. Injector and detector temperatures were 250°C. Spectra were compared with the NIST library and FA mass spectra archive (The AOCS Lipid Library 2015). The assay was performed three times on separate days.

### Assessment of genetic changes in the *S. aureus* 5812R strain

#### DNA extraction, genome sequencing, and assembly

Genomic DNA was isolated from overnight broth culture using Monarch HMW DNA Extraction Kit (New England Biolabs, USA). DNA concentration was estimated using Qubit 4.0 fluorimeter (Thermo Scientific, Germany). DNA quality was evaluated using NanoPhotomete N120 spectrophotometer (Implen, Germany). Genomic libraries were constructed using the NEBNext Ultra II FS library preparation kit (New England Biolabs, USA) according to the manufacturer's instruction for the MiSeq (Illumina, US) sequencing. Sequencing was carried out in the Center of Shared Scientific Equipment “Microorganisms Persistence” of the Institute of Cellular and Intracellular Symbiosis of the Ural Branch of the Russian Academy of Sciences using a MiSeq reagent kit V3 with 2×300-bp reads.

Nanopore sequencing libraries were generated using the Native Barcoding Kit SQK-NBD112.24 (Oxford Nanopore Technologies, UK) protocol and sequenced on a SpotON flow cell vR9 (catalog number FLO-MIN106) for 48 h.

The quality of the raw Illumina reads was analyzed by FastQC software (Andrews, 2010). Trimming of the raw reads was done by Trimmomatic v. 0.39 software (Bolger et al., 2014). Reads were filtered by minimum quality (Q = 20) and length (removing 10 nucleotides from both ends). The obtained nanopore long reads were trimmed for minimal length (5,000 bp) and quality (q = 9) with Nanofilt (Coster et al., 2018). Genome assembly was performed using Unicycler v.0.4.9b (Wick et al., 2017) in normal mode. The draft assembly was then polished using error-corrected Illumina reads with Pilon 1.23 (Walker et al., 2014).

#### Annotation and subsystem analysis

The annotation of genome was carried out using the default Prokka pipeline (https://github.com/tseemann/prokka). The predicted CDSs were annotated based on COG (Cluster of Orthologous Groups of proteins) and KEGG (Kyoto Encyclopedia of Gene and Genomes) (Kanehisa and Goto, 2000) databases, using eggNOG 5.0 (Huerta-Cepas et al., 2019) and KASS (Moriya et al., 2007), respectively. A graphical circular map of the genome was performed with the CGview server (Grant and Stothard, 2008).

##### SNP identification

Identification of SNP was performed using the Snippy software (https://github.com/tseemann/snippy). The fastq clean reads of resistant strain *S. aureus* 5812R were aligned against complete genome of wild-type strain *S. aureus* FDA209P.

#### RNA extraction and real-time quantitative PCR analysis

Bacterial strains were grown in Mueller Hinton Broth in the absence of gausemycin A. *S. aureus* FDA209P and *S. aureus* 5812R cells were collected at mid-exponential, late-exponential, and stationary growth phases and diluted to an optical density corresponding to 10^6^ CFU/ml.

Total RNA was isolated using the RNeasy Mini kit (Qiagen, US) according to the manufacturer's instructions. Total RNA was further treated with DNase I (New England Biolabs, US) followed by the RNeasy MinElute Cleanup kit (Qiagen, US) according to the manufacturer's instructions. RNA was quantified using Qubit 4.0 (Thermo Fisher Scientific, US) and the quality of the extracted RNA was assessed by TapeStation 4150 (Agilent Technologies, US). Samples with preserved 16S and 23S peaks and RIN values >8 were selected for gene expression analyses. The complementary DNA was obtained using the iScript reversed transcription supermix for RT-qPCR reagent (Bio-Rad, US) in accordance with the manufacturer's protocol. Then quantitative PCR was carried out using SsoAdvanced Universal SYBR Green Supermix reagent (Bio-Rad, US). Each reaction mixes with a volume of 20 μL was prepared with 300 nM each primer (final concentration) and 20 ng of template RNA.

LightCycler96 Real-Time PCR detection system (Roche, Switzerland) was used with the following thermal cycling conditions: denaturation at 95^0^C for 1 min, followed by 40 cycles of denaturation at 95^0^C for 10 s and annealing/extension at 60^0^C for 15 s. After the last amplification cycle, a melting curve analysis was carried out by heating from 65 to 95 ^0^C in increments of 0.5^0^C /s. Negative controls (without template or reverse transcriptase enzyme) were included in each run.

The design of oligonucleotide primer sequences for the gene responsible for the synthesis of cardiolipin (*cls*) was carried out using the online service “Integrated DNA technologies” (https://www.idtdna.com/Primerquest/Home/Index) using gene sequences present in the GenBank database. Fold changes in the gene expression levels were normalized in relation to the levels of *gyr*B mRNA (Supplementary Table S1).

The relative changes in gene expression were quantified using the Pfaffl method (Pfaffl, 2001):


$$\begin{array}{l} \text{gene expression ratio}={\left(E_{\text{target}}\right)}^{\Delta \text{Cttarget}\left(\text{control}-\text{sample}\right)}\\ \;/{\left(E_{\text{reference}}\right)}^{\Delta \text{Ctreference}\left(\text{control}-\text{sample}\right)}, \end{array}$$

where *E*_*target*_ is the amplification efficiency of the target (gene of interest); *E*_*reference*_ is the amplification efficiency of reference (*gyrB*); *Ct* is the point at which the fluorescence rises above the background fluorescence; Δ*Ct target* is the Ct deviation of the control minus the sample of the target gene transcript; and ΔCt reference is the Ct deviation of the control minus the sample of the reference gene transcript.

### Checkerboard technique

The effect of gausemycin A in combination with other antibiotics was evaluated using the checkerboard technique with modifications (Nwabor et al., 2021). Briefly, 80 μL of diluted bacterial suspension (10^6^ CFU/mL) was added to wells containing 10 μL of sub-inhibitory concentrations of gausemycin A and 10 μL of sub-inhibitory concentrations of conventional antibiotics. The plates were incubated for 24 h at 37°C. The effects of the antimicrobial combination were defined according to the fractional inhibitory concentration index (FICI). The ΣFICs were calculated as follows:

ΣFIC = FIC A + FIC B,

where FIC A is the MIC of drug A in the combination/MIC of drug A alone; and FIC B is the MIC of drug B in the combination/MIC of drug B alone. The result was interpreted as FIC ≤ 0.5 = Synergism, FIC > 0.5 to 1 = additivity, FIC >1 to 2 = indifference, and FIC >2 antagonism.

### Statistical processing

The obtained results were statistically manipulated using Origin 2021 (OriginLab Corporation, Northampton, MA, USA) software. The pair-sample Student's *t*-test was used to estimate the significance of the differences between the pairs.

## Results

### The dynamics of the resistance formation

The first goal of this work is to evaluate the dynamics of the resistance development of *S. aureus* FDA209P to gausemycin A in comparison with ramoplanin and daptomycin, which are similar in structure and mechanism of action, respectively.

The resistant population of *S. aureus* FDA209P was obtained by multiple serial passages with sub-inhibitory concentrations of gausemycin A. The formation of the resistant variant of *S. aureus* FDA209P was established within 20 passages (Table 1).

**Table 1:** The serial passaging of *S. aureus* FDA209P in increasing concentrations of antimicrobials.

**Passage #**	**The highest concentration of antibiotic** **allowing growth (**μ**g/mL)**		
	**Gausemycin A**	**Daptomycin**	**Ramoplanin**
1	2.5	1.25	3.12
2	2.5	1.25	6.25
3	5	1.25	6.25
4	5	1.25	12.5
5	5	1.25	50
6	5	1.25	50
7	5	1.25	50
8	10	1.25	100
9	20	1.25	100
10	40	1.25	100
11	40	1.25	>100
12	40	2.50	>100
13	50	2.50	>100
14	100	2.50	>100
15	100	2.50	>100
16	100	2.50	>100
17	150	2.50	>100
18	200	2.50	>100
19	200	2.50	>100
20	>200	5.0	>100

From the 12th passage with gausemycin A, the bacteria became significantly less sensitive. The obtained clone of *S. aureus* FDA209P at the 13th passage was resistant to the antibiotic concentration in the medium equal to 50 μg/mL.

The adapted population was found to display up to 80-fold increment in their minimum inhibitory concentration (MIC) relative to the wild-type strain. *S. aureus* FDA209P developed resistance to ramoplanin within 10 passages. Right from the 5th passage of cultivation with ramoplanin, the bacteria became significantly less sensitive to this antibiotic. Eight passages were enough to reach MIC value for 100 μg/mL. At the same time, the increase in resistance of *S. aureus* FDA209P to daptomycin during the series of passages was less pronounced. Daptomycin‘s minimum inhibitory concentration (MIC) increased from 1.25 μg/mL to 5.0 μg/mL due to 20 passages (Table 1).

### Cross-resistant of *S. aureus* 5812R

The cross-resistance of the obtained *S. aureus* 5812R strain was evaluated against the main class of antibiotics targeting Gram-positive bacterial barrier structures (cell wall and/or cytoplasmic membrane). Antibiotics with other targets of action were also used, including those affecting the 30S subunit of the ribosome and inhibiting bacterial DNA topoisomerases and gyrase.

It was found that the most significant difference was acquired in susceptibility to daptomycin, which was 4-fold more potent against *S. aureus* FDA209P compared to *S. aureus* 5812R. Surprisingly, the two compared strains did not differ in their susceptibility to vancomycin, ramoplanin, and nisin (Table 2). MIC values for non-peptide antibiotics changed differently. The most pronounced shift occurred with gentamycin, *S. aureus* 5812R demonstrated phenotype which is 7.8-fold resistant, compared to its parent strain (Table 2). MIC values for cefazolin, tetracycline, and ampicillin increased by twice for *S. aureus* 5812R, while MIC value for ciprofloxacin, on the contrary, decreased twice.

**Table 2:** Susceptibility of *S. aureus* FDA209P and *S. aureus* 5812R to various antimicrobials.

**Compounds**	**MIC**, μ**g/mL**	
	***S. aureus***	***S. aureus***
	**FDA209P**	**5812R**
Gausemycin A	2.5	>200
Ramoplanin	3.12	3.12
Nisin	7.8	7.8
Vancomycin	0.78	0.78
Daptomycin	1.25	5.0
Cefazolin	0.20	0.40
Tetracycline	0.47	0.94
Gentamicin	0.20	1.56
Ciprofloxacin	0.40	0.20
Ampicillin	0.16	1.25

### Phenotype of the resistant clone

The resistance of *S. aureus* 5812R was stable, since multiple (17) passaging throughout the gausemycin A-free media did not change the MIC value for gausemycin A.

**Figure 1:**
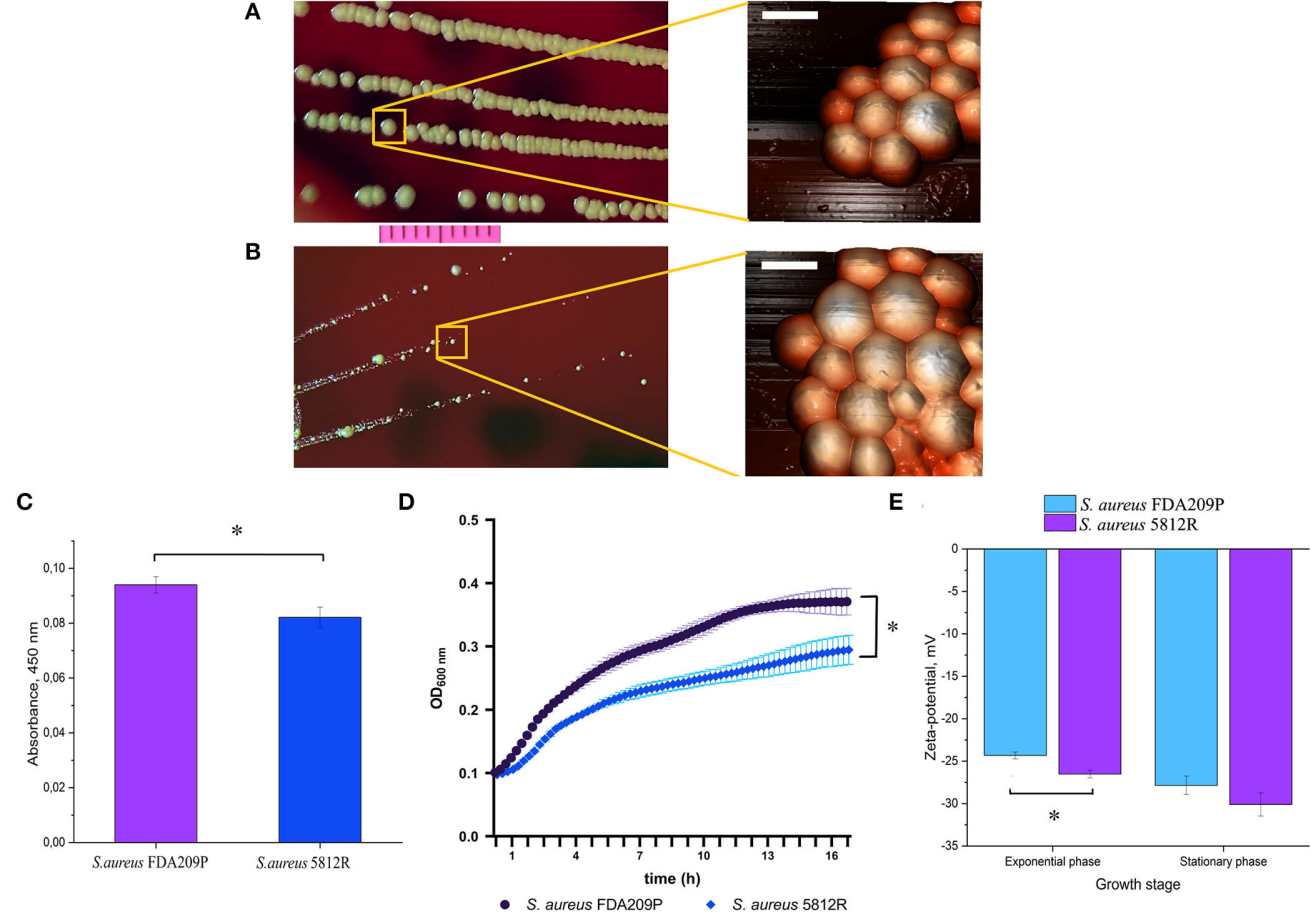
The phenotypical characteristics of the gausemycin A-resistant *S. aureus* 5812R strain. Morphology of colonies and bacterial cells of *S. aureus* FDA209P **(A)** and *S. aureus* 5812R **(B)**, which were formed for 24 h. Optical microscopy (bar-−1 cm) and atomic force microscopy (bar – 1 μm) were used for revealing the morphology of bacterial colonies and cells, respectively. **(C)** The amount of pigment which was extracted from colonies and expressed in absorbance units. The means plus standard deviations of the means of results from three independent experiments with three technical replicates are shown (^*^ *p* < 0.05). **(D)** The growth kinetics of *S. aureus* FDA209P and *S. aureus* 5812R on Muller-Hinton medium without gausemycin A. **(E)** The charges of bacterial cells at various growth stages, which were estimated in zeta-values. The means plus standard deviations of the means of results from two independent experiments with three technical replicates are shown (*p < 0.05).

The phenotype of *S. aureus* 5812R grown on plate resembles a small-colony variant, since it forms colonies a few times smaller than the parent strain (Figures 1A,B), and is less pigmented (Figure 1C) (Proctor et al., 2006; Sulaiman et al., 2021). The AFM revealed no significant difference in cell diameter: 1.001±0.24 μm (*S. aureus* FDA209P) versus 1.0056±0.19 μm (*S. aureus* 5812R) (p-0.9498, Figures 1A,B). The growth rate of *S. aureus* 5812R in liquid medium was compared with the parent strain, but the final cells density was lower (Figure 1D). *S. aureus* 5812R did not differ from its parent strain in their biochemical features (Supplementary Table S2).

### Zeta potential

Zeta potential measurements revealed that the growing *S. aureus* FDA209P population exhibited a slightly more positive charge (−24.32 ± 0.40 mV) than the strain *S. aureus* 5812R (−26.52 ± 0.44 mV). The same relation was obtained for bacterial cells taken from the stationary phase: −27.85 ± 1.08 mV (*S. aureus* FDA209P), and −30.12 ± 1.36 mV (*S. aureus* 5812R) (Figure 1E).

### Membrane integrity assay

**Figure 2:**
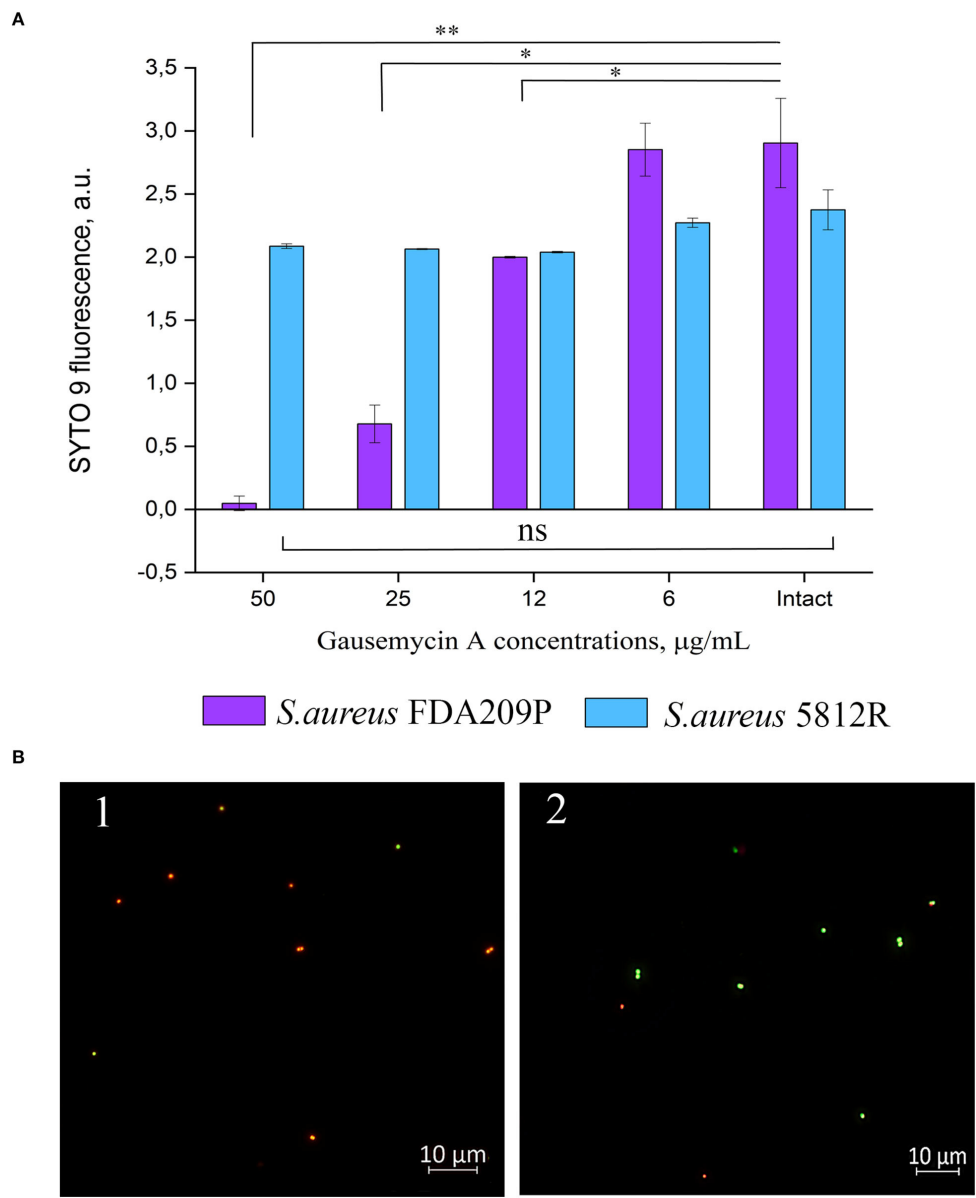
The evaluation of membrane disturbance of *S. aureus* FDA209P and *S. aureus* 5812R using Live/Dead fluorescent dyes. The data show the intensity of SYTO 9 fluorescence at the end-point of measurement (10 min) **(A)**. Epifluorescence microscopy images of *S. aureus* FDA209P (1) and *S. aureus* 5812R (2) after treatment with gausemycin A **(B)**. The means plus standard deviations of the means of results from two independent experiments with three technical replicates are shown (*p < 0.05, **p < 0.01, ns—values are not significantly different).

Our previous study showed that the mechanism of action of gausemycin A (5812-A/C) is through disrupting the structural integrity of bacterial membranes (Vasilchenko et al., 2020). The method of detecting membrane-disturbing action is simple and it is based on the detection of quenching of SYTO9 green fluorescence, when red-fluorescent dye propidium iodide influx through disordered bacterial membrane. If the resistance of staphylococci is associated with a change in the membrane structures of cells, then there will be no quenching of SYTO9 fluorescence. The study showed that adding gausemycin A into the reaction medium with *S. aureus* FDA209P quenched SYTO9 fluorescence in a dose-dependent manner (Figure 2A), and complete quenching was achieved at 50 μg/mL. On the contrary, quenching of SYTO9 fluorescence in the cells of *S. aureus* 5812R was not achieved at all taken concentrations (Figure 2A).

The performed microscopical visualization has revealed that *S. aureus* FDA209P (Figure 2B.1) consisted of numerous cells with permeabilized membranes (glowing red), while cells of *S. aureus* 5812R were predominantly unaffected (glowing green) (Figure 2B.2). Collectively, the obtained data have shown that resistance to gausemycin A is related to membrane modifications.

### Fatty acid profiles of *S. aureus* FDA209P and *S. aureus* 5812R

**Figure 3:**
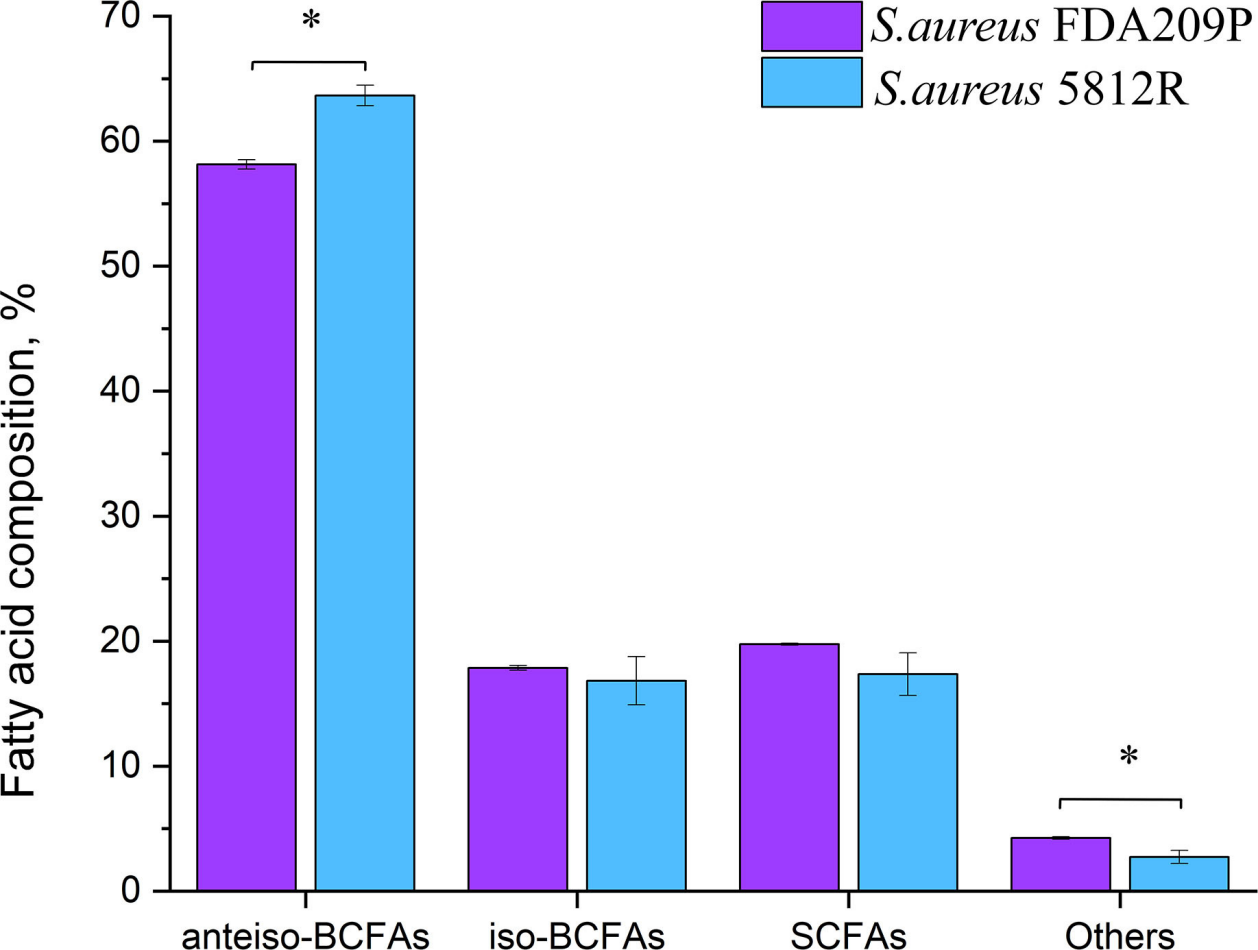
The fatty acid composition of cytoplasmic membrane *S. aureus* FDA209P and *S. aureus* 5812R. The measurements were performed using gas chromatography–mass spectrometry. Y-axis is a relative proportion (%) of each group of fatty acids in relation to the total amount of identified fatty acids. The means plus standard deviations of the means of results from three independent experiments are shown (*p < 0.05).

In total, 26 fatty acids were identified in the extracts of *S. aureus* cells (Table 3). The membrane of *S. aureus* FDA209P is composed by straight chain fatty acids (SCFAs) (17.56 ± 2.03 %) and branched-chain fatty acids (BCFAs) (76.0 ± 0.18 %), including *anteiso*-BCFAs (58.14 ± 0.37%), and *iso*-BCFAs (17.86 ± 0.20%) (Figure 3).

**Table 3:** The membrane fatty acid composition of *S. aureus* cells.

**Lipid numbers**	**Total composition (%** ±**SD)**	
	***S. aureus* FDA209P**	***S. aureus* 5812R**
a-14:0	0.44 ± 0.02	0.44 ± 0.022
a-15:0	33.75 ± 0.34	37.93 ± 1.23
a-17:0	16.15 ± 0.70	19.24 ± 0.67
a-19:0	7.09 ± 0.59	5.40 ± 0.45
a-21:0	0.71 ± 0.16	0.64 ± 0.57
i-15:0	6.42 ± 0.30	6.09 ± 0.79
i-16:0	0.20 ± 0.02	0.72 ± 0.28
i-17:0	5.90 ± 0.12	6.02 ± 1.02
i-18:0	0.66 ± 0.11	0.71 ± 0.023
i-19:0	3.73 ± 0.17	2.69 ± 0.46
i-20:0	0.34 ± 0.04	0.21 ± 0.04
i-21:0	0.62 ± 0.12	0.40 ± 0.11
14:0	0.22 ± 0.03	0.91 ± 0.50
15:0	0.20 ± 0.03	0.30 ± 0.05
16:1	0.14 ± 0.08	0.12 ± 0.09
16:0	3.13 ± 0.30	2.98 ± 0.67
17:0	1.08 ± 0.055	1.11 ± 0.12
18:2	0.11 ± 0.06	0.20 ± 0.06
18:1	0.53 ± 0.19	0.70 ± 0.26
18:0	5.01 ± 0.24	4.60 ± 1.34
19:0	4.24 ± 0.24	3.31 ± 0.37
20:1	0.32 ± 0.04	0.14 ± 0.03
20:0	3.61 ± 0.12	2.47 ± 0.15
21:0	0.57 ± 0.01	0.39 ± 0.13
22:0	0.58 ± 0.29	0.14 ± 0.12
Others	4.25 ± 0.10	2.16 ± 0.52

The membrane of *S. aureus* 5812R consists of insignificantly less amount of SCFAs (17.36 ± 1.07 %, p-0.13239). Comparatively, the decrease of SCFAs in cells of *S. aureus* 5812R was mostly related to C18:0, C19:0, C20:0, and C22:0 fatty acids, which were 0.5–2.0 times less present in comparison with *S. aureus* FDA209P (Table 3). On the contrary, the branched-chain fatty acids (BCFAs) were in prevalence (80.48 ± 1.51 %, p−0.03186), among them there were significantly more *anteiso*-BCFAs (63.64 ± 0.82 %, p−0.0048), whereas the proportion of *iso*-BCFAs was insignificantly less present (16.83±1.93 %, p−0.46085).

Our finding suggests that the change in the proportions of BCFAs in *S. aureus* 5812R is possibly related to the resistance phenomenon.

### Genetic basis of the antibiotic resistance

The DNA sequencing of *S. aureus* FDA209P and *S. aureus* 5812R was performed using both Nanopore sequencing and Next generation sequencing technologies. Performed sequencing of the *S. aureus* FDA209P genome generated 18,875 reads (465,232,550 bases, 167 times coverage) with MinION Mk1B, and 1,849,663 reads (969,265,770 bases, 349 times coverage) with MiSeq. At the same time, for the gausemycin A-resistant *S. aureus* 5812R strain, 15,814 reads (394,758,410 bases, 142 times coverage) and 1,998,645 reads (1,047,191,454 bases, 377 times coverage) were obtained by MinION and MiSeq, respectively (Supplementary Table S3). The assembled genomes were released in GenBank NCBI for further study and annotation (*S. aureus* FDA209P JANQDW000000000; *S. aureus* 5812R JANPYH000000000).

About 1512 (59.4%) annotated protein-coding genes were classified according to KEGG categories. The most represented functional identified categories were genetic information processing (359), carbohydrates (178), signaling and cellular processing (169), environmental information processing (129), metabolism of cofactors and vitamins (87), amino acids metabolism (84), nucleotide metabolism (64), and energy metabolism (45). Among 2,547 protein-coding genes, seven genes are associated with the resistance to cationic antimicrobial peptides (CAMPs) (Supplementary Table S4).

**Figure 4:**
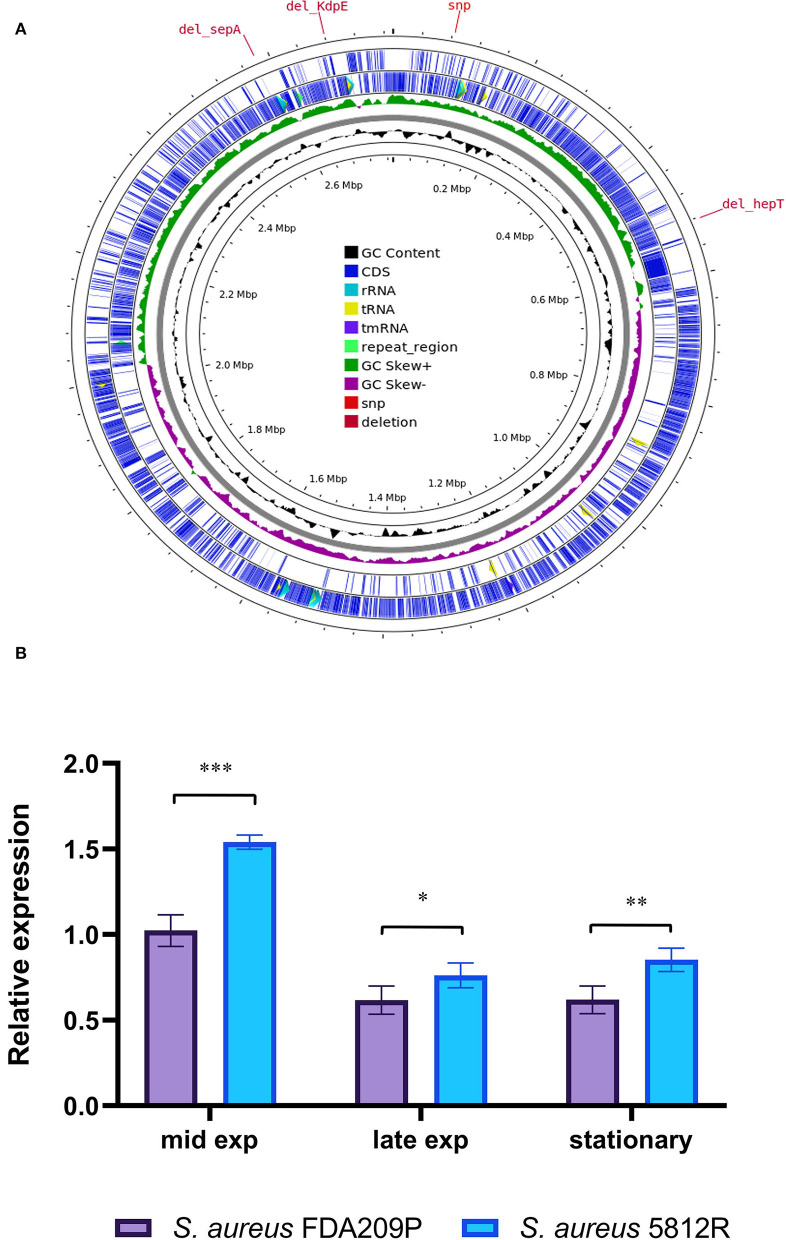
Graphical circular map of the *S. aureus* 5812R chromosome **(A)**. The map was generated using CGView Server (http://cgview.ca/). The expression analysis of gene responsible for the synthesis of cardiolipin (*cls*) **(B)**. Transcript levels of the analyzed genes were determined by RT-qPCR in relation to the *gyrB* expression. Mid Exp—the mid-exponential growth phase; Late Exp—the late exponential growth phase; Stationary—the stationary growth phase. The means plus standard deviations of the means of results from three technical replicates are shown (*p < 0.05, **p < 0.01, ***p < 0.001).

Comparing the nucleotide sequences of both strains, we have found one single nucleotide polymorphism (SNP) and deletions of three genes in *S. aureus* 5812R genome (Table 4, Figure 4A). SNP was non-synonymous and it was found in the *vraT* gene (previously called *yvqF*) (C → G at the nucleotide 370), which encodes the membrane protein with unknown biological function. The *hepT* gene, encoding heptaprenyl diphosphate synthase component II has the single nucleotide deletion at position 940. The *sepA* gene, which encodes the multidrug-resistant efflux pump protein SepA, has five nucleotide deletions between 347-351. This mutation has caused a frameshift in *sepA* and—presumably—premature termination. Also, the sequencing revealed deletions of five nucleotides between 585 and 589 positions in the *kdpE* gene, which is a transcriptional activator in the two-component system KdpD/KdpE.

**Table 4:** Genetical differences between the wild-type *S. aureus* FDA209P and its mutant variant *S. aureus* 5812R.

**Nucleotide position in *S. aureus* FDA209P**	**Type of mutation**	***S. aureus* FDA209P**	***S. aureus* 5812R**	**nucleotide position in the gene**	**amino acid position**	**Effect**	**Gene**
83,211	snp	C	G	370/702	124/233	missense variant c.370 C>G p. Arg124Gly	*vraT*
521,611	del	GA	G	940/960	314/319	frameshift variant c.940delA p. Met314fs	*hepT*
2,581,132	del	GTTATT	G	347/474	116/157	frameshift variant c.347_351, del TTATT p. Phe116fs	*sepA*
2,679,574	del	ATATGC	A	589/696	195/231	frameshift variant c.585_589, del GCATA p. His196fs	*kdpE*

### The comparison of cardiolipin synthase (*Cls*2) gene expression with *S. aureus* FDA209P and *S. aureus* 5812R

Cardiolipin plays a critical role in bacterial physiology, especially for cytokinesis and antibiotic resistance (Tiwari et al., 2020). The synthesis of cardiolipin in bacteria is catalyzed by the cardiolipin synthases Cls, which provide the condensation of two phosphatidylglycerol molecules to yield cardiolipin and glycerol. We analyzed the expression of the *cls2* (cardiolipin synthase) gene in both strains in a comparison manner.

It was found that the most active expression of the *cls2* gene occurred in the mid-log phase of bacterial growth. Continued growth was associated with a double decrease in the expression level. The difference between the compared strains was a higher level of *cls2* expression in the resistant strain 5812R. In the mid-log phase, the relative expression of the *cls2* gene was higher in *S. aureus* 5812R cells (1.54 ± 0.04), compared to *S. aureus* FDA209P (1.02 ± 0.06, p-0.00115). The following bacterial growth was accompanied by the reduction in the *cls2* gene relative expression. In the late-exponential phase, the relative expression decreased to 0.61 ± 0.03 (*S. aureus* FDA209P) and 0.76 ± 0.05 (*S. aureus* 5812R) (p-0.03876); in the stationary phase, the relative expression was 0.62 ± 0.05 for *S. aureus* FDA209P, and 0.85 ± 0.04 for *S. aureus* 5812R (p−0.00985) (Figure 4B).

Thus, we showed that gausemycin A resistance is accompanied by the increased relative expression of the *cls2* gene in the *S. aureus* 5812R cells.

### The antibacterial effect of gausemycin A in combination with conventional antibiotics

The antibacterial activity of four combinations of antibiotics against *S. aureus* 5812R strain was tested using the checkerboard method. The effects of combinations of antimicrobials were determined by the index of fractional inhibitory concentration (FICI). The results of *in vitro* antibacterial activity of gausemycin A in combination with other antibiotics against *S. aureus* 5812R are presented in Table 5. Three antibiotic combinations showed a synergistic activity resulting in the reduction in MICs. The combination of Cip + Gau showed a pronounced synergistic antibacterial effect against *S. aureus* 5812R (FICI 0.49).

**Table 5:** The *in vitro* antibacterial activity of gausemycin A in the combination with other antibiotics against *S. aureus* 5812R.

**Strain**	**MIC alone (**μ**g/ml)**					**The effect of antibiotics in combination**											
	**GauA**	**Dap**	**Amp**	**Cip**	**Gen**	**Cip**+ **GauA**			**Dap** + **GauA**			**Amp** + **GauA**			**Gen**+ **GauA**		
						**MIC (**μ**g/ml)**		**FICI**	**MIC (**μ**g/ml)**		**FICI**	**MIC (**μ**g/ml)**		**FICI**	**MIC (**μ**g/ml)**		**FICI**
						**Cip**	**GauA**		**Dap**	**GauA**		**Amp**	**GauA**		**Gen**	**GauA**	
*S. aureus* 5812R	>150	5.0	1.25	0.20	1.56	0.18	2.3	0.49	>12	>150	3.40	0.63	12.5	0.63	0.75	75	0.98

FIC ≤ 0.5 = Synergy; FIC > 0.5 to 1 = additivity, FIC >1 to 2 = indifference, FIC >2 antagonism; GauA, Gausemycin A; Dap, Daptomycin; Amp, Ampicillin; Cip, Ciprofloxacin; Gen, Gentamicin.

The combinations of Amp + Gau (FICI 0.63) and Gen + Gau (FICI 0.98) showed an additive effect. The combination of Dap + Gau demonstrated an antagonistic effect (FICI 3.40).

## Discussion

Natural lipoglycopeptides are rare and are grouped in a small number of structural classes including gausemycins (Freitas et al., 2022). The present work is the only second evidence of bacterial resistance to lipoglycopeptides after Schmidt's study of ramoplanin-resistant *S. aureus* (Schmidt et al., 2010). Ramoplanin and daptomycin are peptide antibiotics, which are similar in structure and/or mechanism of action, and also are available as commercial drugs. The obtained results indicate that the development of the resistance to gausemycin A was much faster than to daptomycin, but much slower than to ramoplanin.

Since the structure of gausemycin A molecule is unique, it would be interesting to determine whether the *S. aureus* 5812R strain would be cross-resistant to the closest analogs and other antibiotics. It was found that the resistance to gausemycin A does not provide resistance to nisin, vancomycin, and ramoplanin. These peptide antibiotics are united together by the same target within bacterial cells. For example, nisin has a membrane-destroying effect upon binding to a receptor, which is lipid II (Paiva et al., 2011). Vancomycin is a cationic glycopeptide that kills bacteria by binding to the C-terminal D-Ala–D-Ala residues of the peptidoglycan precursor lipid II, and prevents the use of the precursor for cell wall synthesis (Hu et al., 2016). The pyrophosphate pocket of lipid II is also the target for ramoplanin (Cheng et al., 2014).

Our primary objective was to find out what kind of changes could be responsible for staphylococcal resistance to gausemycin A. First of all, it was necessary to evaluate the changes at the genome level. The comparison of *S. aureus* FDA209P and *S. aureus* 5812R genomes revealed deletions. Among the mutations, *hepT* and *kdpE* are the genes which could have the most contribution in gausemycin A resistance.

The *kdpE* gene is a transcriptional activator that is a part of the two-component system KdpD/KdpE (Kdp system) (Freeman et al., 2013). The Kdp system has a function in *S. aureus* as a K^+^ uptake system that directly regulates virulence and stress response. KdpE can bind directly to the DNA promoters of a range of virulence genes. It was found that deletion of *kdpD/kdpE* altered the level of transcription of over 100 genes (Zhao et al., 2010). Existing studies link *kdpD/kdpE* with resistance to antimicrobial stresses, since gene knockouts increase bacterial susceptibility to polymyxins and chitosan, compared to the wild strains (Alegado et al., 2011; Mellegård et al., 2011). Our results suggest further studies to uncover the role of the Kdp system in bacterial resistance to peptide antibiotics.

The resistance of *S. aureus* 5812R to gausemycin A, as well as to daptomycin and other antibiotics could be explained by the transition of cells into the special phenotype, known as a small-colony variant (SCV), that is a variant subpopulation of *S. aureus* characterized by the deficiency in electron transport, and the deficiency in thymidine biosynthesis (Proctor et al., 1998; Watkins et al., 2012; Johns et al., 2015). The reduced electrochemical gradient in SCV cells promotes resistance to gentamycin and other antibiotics (Proctor et al., 1998). The prerequisite for this was found in the analysis of genetic differences between the *S. aureus* FDA209P and the *S. aureus* 5812R strains. Sequencing revealed single nucleotide deletion in the gene *hepT* which encodes protein associated with the isoprenoid biosynthesis pathway (Liu et al., 2016). Isoprenoids are involved in the production of carotenoids, quinones, hemes, and other compounds, which are necessary for the development of normal colony morphotypes (Liu et al., 2016). Deletions in *hepT* lead to blockage in menaquinone biosynthesis (Wakeman et al., 2012). Thus, this nucleotide deletion could be associated with the transition of the *S. aureus* population to the SCV morphotype. The genome analysis also gives a possible explanation why *S. aureus* 5812R is more susceptible to ciprofloxacin than the parent strain. Nucleotide deletions in the *sepA* gene could be associated with the susceptibility of *S. aureus* 5812R to ciprofloxacin, since SepA is an efflux pump that allows bacteria to be resistant to this antibiotic (Hassanzadeh et al., 2017).

Our secondary objective was to find the phenotypic changes that are associated with staphylococcal resistance to gausemycin A. We evaluated the structural changes that occurred with the cytoplasmic membrane, since it is the main target for gausemycin A (Vasilchenko et al., 2020). The bacterial membrane equilibrium between rigidity and fluidity is driven by multiple factors that impact membrane order (water, metal ions, proteins, carotenoids, staphyloxanthin, etc.), among these factors the composition of phospholipid fatty acids is the main factor. (Boudjemaa et al., 2018).

In the case of *S. aureus*, bacterial membranes mainly consist of straight-chain (SCFAs) and branched-chain (BCFAs) saturated fatty acids. SCFAs pack together to produce a bilayer with low permeability properties, while branched-chain *iso* or/and *anteiso* methyl species promote a more fluid membrane structure (Zhang and Rock, 2008). The development of resistance of *S. aureus* 5812R to gausemycin A has been accompanied by an increase in the relative proportion of *anteiso*-BCFAs in cell membranes and by a decrease in the level of *iso*-BCFAs. At the same time, the percentage of saturated fatty acids in the cell membrane of the resistant strain decreased slightly compared to the parent strain. This result is inconsistent with previous studies showing that *S. aureus* responded to higher concentrations of daptomycin and vancomycin by lowering the biosynthesis of *iso* and *anteiso* branched-chain fatty acids while increasing the levels of saturated fatty acids (Mirani and Jamil, 2013; Boudjemaa et al., 2018).

The main *S. aureus* membrane lipids consist negatively charged phospholipids: phosphatidylglycerol (PG) and cardiolipin (CL) (Jung et al., 2008). Cardiolipin synthases (Cls) are the critical enzymes for the synthesis of CL in bacteria. It is known that mutation in *csl2* gene increases daptomycin susceptibility in *S. aureus* (Peleg et al., 2012), while the enhanced levels of cardiolipin are associated with daptomycin resistance in Enterococci (Palmer et al., 2011).

We found out that the *S. aureus* 5812R strain showed an increased relative expression of the *cls2* gene. It seems that cardiolipin plays a more important role in the resistance of staphylococci to gausemycin A, than daptomycin which could be explained by the differences in their physicochemical properties. Gausemycin A molecule has a more negative net charge compared to daptomycin. Zeta potential measurements revealed the *S. aureus* FDA209P population consisted of cells that are slightly more positively charged compared to the *S. aureus* 5812R population. Although the difference is not high, it may contribute to the resistance to gausemycin A.

Thereby, antimicrobial peptides are increasingly recognized as a promising class of compounds that are used in combination with conventional antibiotics. In this regard, we have tried to overcome gausemycin A resistance by choosing that combination, where a pair of antibiotics affects another cellular target, for example, DNA replication or protein synthesis. Besides, we tested the combination of gausemycin A and daptomycin, and, surprisingly, revealed the antagonism between them for an unknown reason. Similar work showed that the combinations of daptomycin with other peptide antibiotics resulted in indifferent interactions, while the combination of daptomycin + ciprofloxacin had a synergistic effect against *S. aureus* (Mataraci and Dosler, 2012). Numerous studies demonstrated the effectiveness of the combination of daptomycin with antibiotics, which target nucleic acid replication (Rand and Houck, 2004; Kelesidis et al., 2011; Kamble et al., 2022). In our study, the combination of ciprofloxacin and gausemycin A has been found to have synergistic activity against gausemycin A-resistant *S. aureus*. Thus, it has been revealed that such a combination could be successful to suppress the gausemycin A-resistant *S. aureus*.

## Conclusion

In this study, we showed the ability of *S. aureus* FDA209P to form gausemycin A-resistant phenotype. However, the revealed phenomenon does not mean that this peptide antibiotic has no prospects as a therapeutic agent. Ramoplanin showed an even more pronounced ability to form a resistant *S. aureus* phenotype in this comparative experiment. In addition, *S. aureus* 5812R did not form significant cross-resistance to antibiotics that other cellular targets have, which allows considering gausemycin A in combination therapy using conventional antibiotics like ciprofloxacin.

The obtained results also deal with the mechanism of action and the bacterial response of gausemycin A. We found that *S. aureus* 5812R was cross-resistant to daptomycin. This finding confirms the similarity between gausemycin A and daptomycin in their modes of action. At the same time, the identified features of genetic and phenotypic changes associated with bacterial resistance suggest that the response to gausemycin A is specific due to the uniqueness of the structure of gausemycin A.

## Data availability statement

The original contributions presented in the study are included in the article/Supplementary materials, further inquiries can be directed to the corresponding author.

## Author contributions

DP, ER, SK, and AV conducted the experiments. DP, SK, AV, VS, and OL performed analyses. DP, ER, and AV wrote the manuscript and prepared the figures. AV conceived, designed the experiments, and edited the manuscript. All authors contributed to the article and approved the submitted version.

## Funding

This work was supported by the Russian Science Foundation (project number 21-74-00123).

## Conflict of interest

The authors declare that the research was conducted in the absence of any commercial or financial relationships that could be construed as a potential conflict of interest.

## Publisher's note

All claims expressed in this article are solely those of the authors and do not necessarily represent those of their affiliated organizations, or those of the publisher, the editors and the reviewers. Any product that may be evaluated in this article, or claim that may be made by its manufacturer, is not guaranteed or endorsed by the publisher.
