# Supplementary material for: Staphylococcus aureus is able to generate resistance to novel lipoglycopeptide antibiotic gausemycin A
Source: Front Microbiol. 2022 Sep 29;13:963979. doi: 10.3389/fmicb.2022.963979 (PMC9558223; doi:10.3389/fmicb.2022.963979)
Supplement: Supplementary file 1 [file Table_1.DOCX]

***Staphylococcus aureus* is able to Generate Resistance to Novel Lipoglycopeptide Antibiotic Gausemycin A**

Darya V. Poshvina^1^, Diana S. Dilbaryan^1^, Sergey P. Kasyanov^4^, Vera S. Sadykova^3^, Olda A. Lapchinskaya^3^, Eugene A. Rogozhin^2, 3^and Alexey S. Vasilchenko^1*^

^1^Laboratory of Antimicrobial Resistance, Institute of Environmental and Agricultural Biology (X-BIO), Tyumen State University, Tyumen, Russia

^2^Shemyakin and Ovchinnikov Institute of Bioorganic Chemistry Russian Academy of Sciences, Moscow, Russia

^3^Gause Institute of New Antibiotics, Moscow, Russia

^4^A.V. Zhirmunsky National Scientific Center of Marine Biology, Vladivostok, Russia

*Corresponding author at: Institute of Environmental and Agricultural Biology (X-BIO), Tyumen State University, 625003, 23 Lenina St., Tyumen, Russian Federation

E-mail addresses: [avasilchenko@gmail.com](mailto:avasilchenko@gmail.com) (**Alexey S. Vasilchenko)**.

Table S1. The primers used in the RT-qPCR study

| **Gene description (designation)** | **Primer sequence** |
| --- | --- |
| DNA gyrase subunit B (*gyr*B) | F: GACGTGGTATCCCAGTTGATATT |
|  | R: ACCATGTAAACCACCAGATACTT |
| Cardiolipin synthase (*cls*) | F: TGGGCATGGCTATTTGTACTT |
|  | R: TCAATTTGCGTGCCGAAAC |

Table S2. The biochemical features of wild strain *S.aureus* FDA209P and its resistant mutant *S.aureus* 5812R

| **Substrates** | **Enzymes** | ***Staphylococcus aureus* FDA209 P** | ***Staphylococcus aureus* 5812R** |
| --- | --- | --- | --- |
| urea | Urease | Positive | Positive |
| L-arginine | Arginine Dihydrolase | Positive | Positive |
| L-ornithine | Ornithine Decarboxylase | Negative | Negative |
| Esculin ferric citrate | Hydrolysis (Esculin) | Positive | Positive |
| D-glucose | Fermentation (Glucose) | Intermediate * | Positive |
| D-fructose | Fermentation (Fructose) | Positive | Positive |
| D-mannose | Fermentation (MannosE) | Positive | Positive |
| D-maltose | Fermentation (MalLtose) | Positive | Positive |
| D-lactose (bovine origin) | Fermentation (Lactose) | Positive | Positive |
| D-trehalose | Fermentation (Trehalose) | Positive | Positive |
| D-mannitol | Fermentation (Mannitol) | Positive | Positive |
| D-raffinose | Fermentation (Raffinose) | Negative | Negative |
| D-ribose | Fermentation (Ribose) | Intermediate * | Positive |
| D-cellobiose | Fermentation (Cellobiose) | Negative | Negative |
| Potassium nitrate | Reduction (Nitrates) | Negative | Negative |
| Sodium pyruvate | Acetoin production (Voges Proskauer) | Negative | Negative |
| 2-naphthyl-βD-galactopyranoside | β-galactosidase | Negative | Negative |
| L-arginine β-naphthylamide | Arginine Arylamidase | Negative | Negative |
| 2-naphthyl phosphate | Alkaline phosphatase | Negative | Negative |
| Pyroglutamic acid-β-naphthylamide | Pyrrolidonyl Arylamidase | Negative | Negative |
| novobiocin | Resistance (Novobiocin) | Positive | Positive |
| D-saccharose (sucrose) | Fermentation (Saccharose) | Positive | Positive |
| N-acetyl-glucosamine | Fermentation (N-acetyl-Glucosamine) | Positive | Positive |
| D-turanose | Fermentation (Turanose) | Positive | Positive |
| L-arabinose | Fermentation (Arabinose) | Negative | Negative |
| 4-nitrophenyl-βD-glucuronide | Β GlucuRonidase | Negative | Negative |

* result is not clear

Table S3. Sequencing statistic of *S. aureus* libraries

| **Strain** | **Raw Illumina reads** | **Filtered Illumina reads** | **Raw Nanopore bp** | **Filtered Nanopore bp** | **Total coverge** |
| --- | --- | --- | --- | --- | --- |
| *S_aureus* FDA209P | 1,849,663 | 1,822,497 | 1,201,697,641 | 465,232,550 | ×516 |
| *S.aureus* 5812R | 1,998,645 | 1,968,833 | 1,016,742,999 | 394,758,410 | ×519 |

Table S4. Statistic of genome features of bacterial strains used in this study

| **Genome information** | ***S. aureus* FDA209P** | ***S. aureus* 5812R** |
| --- | --- | --- |
| GenBank accession number | JANQDW000000000 | JANPYH000000000 |
| Genome size, bp | 2772454 | 2772443 |
| Number of contigs | 1 | 1 |
| GC content | 32.88% | 32.88% |
| Protein-coding sequences | 2547 | 2547 |
| rRNAs | 19 | 19 |
| tRNAs | 61 | 61 |
| tmRNA | 1 | 1 |
| CRISPR arrays | 2 | 2 |
